# Supplementary material for: Risk factors for new-onset atrial fibrillation in patients with chronic obstructive pulmonary disease: a systematic review and meta-analysis
Source: PeerJ. 2020 Dec 2;8:e10376. doi: 10.7717/peerj.10376 (PMC7718784; doi:10.7717/peerj.10376)
Supplement: Supplemental Information 1 [file peerj-08-10376-s001.docx]

# Supplementary

**Appendix 1.** Search Strategy

1. **Pubmed**

Search **(((((((((Atrial fibrillation*[Title/Abstract]) OR Atrial flutter*[Title/Abstract]) OR Auricular fibrillation*[Title/Abstract])) OR (((((((Atrial Flutters[Title/Abstract]) OR Flutter, Atrial[Title/Abstract]) OR Flutters, Atrial[Title/Abstract]) OR Auricular Flutter[Title/Abstract]) OR Auricular Flutters[Title/Abstract]) OR Flutter, Auricular[Title/Abstract]) OR Flutters, Auricular[Title/Abstract])) OR "Atrial Flutter"[Mesh]) OR (((((((((((((((((((((((((Atrial Fibrillations[Title/Abstract]) OR Fibrillation, Atrial[Title/Abstract]) OR Fibrillations, Atrial[Title/Abstract]) OR Auricular Fibrillation[Title/Abstract]) OR Auricular Fibrillations[Title/Abstract]) OR Fibrillation, Auricular[Title/Abstract]) OR Fibrillations, Auricular[Title/Abstract]) OR Persistent Atrial Fibrillation[Title/Abstract]) OR Atrial Fibrillation, Persistent[Title/Abstract]) OR Atrial Fibrillations, Persistent[Title/Abstract]) OR Fibrillation, Persistent Atrial[Title/Abstract]) OR Fibrillations, Persistent Atrial[Title/Abstract]) OR Persistent Atrial Fibrillations[Title/Abstract]) OR Familial Atrial Fibrillation[Title/Abstract]) OR Atrial Fibrillation, Familial[Title/Abstract]) OR Atrial Fibrillations, Familial[Title/Abstract]) OR Familial Atrial Fibrillations[Title/Abstract]) OR Fibrillation, Familial Atrial[Title/Abstract]) OR Fibrillations, Familial Atrial[Title/Abstract]) OR Paroxysmal Atrial Fibrillation[Title/Abstract]) OR Atrial Fibrillation, Paroxysmal[Title/Abstract]) OR Atrial Fibrillations, Paroxysmal[Title/Abstract]) OR Fibrillation, Paroxysmal Atrial[Title/Abstract]) OR Fibrillations, Paroxysmal Atrial[Title/Abstract]) OR Paroxysmal Atrial Fibrillations[Title/Abstract])) OR "Atrial Fibrillation"[Mesh])) AND ((((((((("Pulmonary Disease, Chronic Obstructive"[Mesh]) OR (((((((((COPD[Title/Abstract]) OR Chronic Obstructive Pulmonary Disease[Title/Abstract]) OR COAD[Title/Abstract]) OR Chronic Obstructive Airway Disease[Title/Abstract]) OR Chronic Obstructive Lung Disease[Title/Abstract]) OR Airflow Obstruction, Chronic[Title/Abstract]) OR Airflow Obstructions, Chronic[Title/Abstract]) OR Chronic Airflow Obstructions[Title/Abstract]) OR Chronic Airflow Obstruction[Title/Abstract])) OR "Lung Diseases, Obstructive"[Mesh]) OR (((((((Lung Disease, Obstructive[Title/Abstract]) OR Obstructive Lung Disease[Title/Abstract]) OR Obstructive Lung Diseases[Title/Abstract]) OR Obstructive Pulmonary Diseases[Title/Abstract]) OR Obstructive Pulmonary Disease[Title/Abstract]) OR Pulmonary Disease, Obstructive[Title/Abstract]) OR Pulmonary Diseases, Obstructive[Title/Abstract])) OR "Pulmonary Emphysema"[Mesh]) OR (((((((((((((((((((((((Emphysemas, Pulmonary[Title/Abstract]) OR Pulmonary Emphysemas[Title/Abstract]) OR Emphysema, Pulmonary[Title/Abstract]) OR Focal Emphysema[Title/Abstract]) OR Emphysema, Focal[Title/Abstract]) OR Emphysemas, Focal[Title/Abstract]) OR Focal Emphysemas[Title/Abstract]) OR Panacinar Emphysema[Title/Abstract]) OR Emphysema, Panacinar[Title/Abstract]) OR Emphysemas, Panacinar[Title/Abstract]) OR Panacinar Emphysemas[Title/Abstract]) OR Panlobular Emphysema[Title/Abstract]) OR Emphysema, Panlobular[Title/Abstract]) OR Emphysemas, Panlobular[Title/Abstract]) OR Panlobular Emphysemas[Title/Abstract]) OR Centriacinar Emphysema[Title/Abstract]) OR Centriacinar Emphysemas[Title/Abstract]) OR Emphysema, Centriacinar[Title/Abstract]) OR Emphysemas, Centriacinar[Title/Abstract]) OR Centrilobular Emphysema[Title/Abstract]) OR Centrilobular Emphysemas[Title/Abstract]) OR Emphysema, Centrilobular[Title/Abstract]) OR Emphysemas, Centrilobular[Title/Abstract])) OR "Bronchitis, Chronic"[Mesh]) OR Chronic Bronchitis[Title/Abstract]))**

1. **The Cochrane Library**

#1 MeSH descriptor: [undefined] explode all trees

#2 (Chronic Obstructive Lung Disease):ti,ab,kw

#3 (Chronic Obstructive Pulmonary Disease):ti,ab,kw

#4 (COPD):ti,ab,kw

#5 (COAD):ti,ab,kw

#6 (Chronic Obstructive Airway Disease):ti,ab,kw

#7 (Airflow Obstruction, Chronic):ti,ab,kw

#8 (Chronic Airflow Obstruction):ti,ab,kw

#9 (Airflow Obstructions, Chronic):ti,ab,kw

#10 (Chronic Airflow Obstructions):ti,ab,kw

#11 #1 or #2 or #3 or #4 or #5 or #6 or #7 or #8 or #9 or #10

#12 MeSH descriptor: [Lung Diseases, Obstructive] explode all trees

#13 (Pulmonary Disease, Obstructive):ti,ab,kw

#14 (Obstructive Pulmonary Diseases):ti,ab,kw

#15 (Obstructive Lung Diseases):ti,ab,kw

#16 (Obstructive Lung Disease):ti,ab,kw

#17 (Lung Disease, Obstructive):ti,ab,kw

#18 (Pulmonary Diseases, Obstructive):ti,ab,kw

#19 (Obstructive Pulmonary Disease):ti,ab,kw

#20 #12 or #13 or #14 or #15 or #16 or #17 or #18 or #19

#21 MeSH descriptor: [Pulmonary Emphysema] explode all trees

#22 (Emphysema, Centrilobular):ti,ab,kw

#23 (Centrilobular Emphysema):ti,ab,kw

#24 (Emphysemas, Pulmonary):ti,ab,kw

#25 (Emphysema, Pulmonary):ti,ab,kw

#26 (Pulmonary Emphysemas):ti,ab,kw

#27 (Emphysema, Panlobular):ti,ab,kw

#28 (Panlobular Emphysema):ti,ab,kw

#29 (Focal Emphysema):ti,ab,kw

#30 (Emphysema, Focal):ti,ab,kw

#31 #21 or #22 or #23 or #24 or #25 or #26 or #27 or #28 or #29 or #30

#32 MeSH descriptor: [Bronchitis, Chronic] explode all trees

#33 (Chronic Bronchitis):ti,ab,kw

#34 #32 or #33

#35 #11 or #20 or #31 or #34

#36 MeSH descriptor: [Atrial Fibrillation] explode all trees

#37 (Atrial Fibrillation, Paroxysmal):ti,ab,kw

#38 (Fibrillation, Paroxysmal Atrial):ti,ab,kw

#39 (Fibrillations, Paroxysmal Atrial):ti,ab,kw

#40 (Atrial Fibrillations, Paroxysmal):ti,ab,kw

#41 (Paroxysmal Atrial Fibrillations):ti,ab,kw

#42 (Paroxysmal Atrial Fibrillation):ti,ab,kw

#43 (Fibrillation, Familial Atrial):ti,ab,kw

#44 (Atrial Fibrillation, Familial):ti,ab,kw

#45 (Familial Atrial Fibrillation):ti,ab,kw

#46 (Fibrillation, Auricular):ti,ab,kw

#47 (Fibrillations, Atrial):ti,ab,kw

#48 (Fibrillations, Auricular):ti,ab,kw

#49 (Fibrillation, Atrial):ti,ab,kw

#50 (Auricular Fibrillations):ti,ab,kw

#51 (Atrial Fibrillations):ti,ab,kw

#52 (Auricular Fibrillation):ti,ab,kw

#53 (Atrial Fibrillation, Persistent):ti,ab,kw

#54 (Fibrillations, Persistent Atrial):ti,ab,kw

#55 (Persistent Atrial Fibrillations):ti,ab,kw

#56 (Persistent Atrial Fibrillation):ti,ab,kw

#57 (Atrial Fibrillations, Persistent):ti,ab,kw

#58 (Fibrillation, Persistent Atrial):ti,ab,kw

#59 #36 or #37 or #38 or #39 or #40 or #41 or #42 #43 or #44 or #45 or #46 or #47 or #48 or#49 or #50 or #51 or #52 or #53 or #54 or #55 or #56 or #57 or #58

#60 MeSH descriptor: [Atrial Flutter] explode all trees

#61 (Flutter, Auricular):ti,ab,kw

#62 (Atrial Flutters):ti,ab,kw

#63 (Flutter, Atrial):ti,ab,kw

#64 (Auricular Flutter):ti,ab,kw

#65 (Flutters, Atrial):ti,ab,kw

#66 (Atrial fibrillation*):ti,ab,kw

#67 (Atrial flutter*):ti,ab,kw

#68 (Auricular fibrillation*):ti,ab,kw

#69 #60 or #61 or #62 or #63 or #64 or #65

#70 #59 or #66 or #67 or #68 or #69

#71 #35 and #70

**3. Web of Science (WOS)**

# 1 TOPIC: (Pulmonary Disease, Chronic Obstructive) OR TOPIC: (COPD) OR TOPIC: (Chronic Obstructive Pulmonary Disease) OR TOPIC: (COAD) OR TOPIC: (Chronic Obstructive Airway Disease) OR TOPIC: (Chronic Obstructive Lung Disease) OR TOPIC: (Airflow Obstruction, Chronic) OR TOPIC: (Airflow Obstructions, Chronic) OR TOPIC: (Chronic Airflow Obstructions) OR TOPIC: (Chronic Airflow Obstruction) OR TOPIC: (Lung Diseases, Obstructive) OR TOPIC: (Lung Disease, Obstructive) OR TOPIC: (Obstructive Lung Disease) OR TOPIC: (Obstructive Lung Diseases) OR TOPIC: (Obstructive Pulmonary Diseases) OR TOPIC: (Obstructive Pulmonary Disease) OR TOPIC: (Pulmonary Disease, Obstructive) OR TOPIC: (Pulmonary Diseases, Obstructive) OR TOPIC: (Pulmonary Diseases, Obstructive)

# 2 TOPIC: (Pulmonary Emphysema) OR TOPIC: (Emphysemas, Pulmonary) OR TOPIC: (Pulmonary Emphysemas) OR TOPIC: (Emphysema, Pulmonary) OR TOPIC:(Focal Emphysema) OR TOPIC: (Emphysema, Focal) OR TOPIC: (Emphysemas, Focal) OR TOPIC: (Focal Emphysemas) OR TOPIC: (Panacinar Emphysema) OR TOPIC:(Emphysema, Panacinar) OR TOPIC: (Emphysemas, Panacinar) OR TOPIC: (Panacinar Emphysemas) OR TOPIC: (Panlobular Emphysema) OR TOPIC: (Emphysema, Panlobular) OR TOPIC: (Emphysemas, Panlobular) OR TOPIC: (Panlobular Emphysemas) OR TOPIC: (Centriacinar Emphysema) OR TOPIC: (Centriacinar Emphysemas) OR TOPIC: (Emphysema, Centriacinar) OR TOPIC: (Emphysemas, Centriacinar) OR TOPIC: (Centrilobular Emphysema) OR TOPIC: (Centrilobular Emphysemas) OR TOPIC: (Emphysema, Centrilobular) OR TOPIC: (Chronic Bronchitis) OR TOPIC: (Bronchitis, Chronic)

# 3 TOPIC: (Atrial Fibrillation) OR TOPIC: (Atrial Fibrillations) OR TOPIC: (Fibrillation, Atrial) OR TOPIC: (Fibrillations, Atrial) OR TOPIC: (Auricular Fibrillation) OR TOPIC:(Auricular Fibrillations) OR TOPIC: (Fibrillation, Auricular) OR TOPIC: (Fibrillations, Auricular) OR TOPIC: (Persistent Atrial Fibrillation) OR TOPIC: (Atrial Fibrillation, Persistent) OR TOPIC: (Atrial Fibrillations, Persistent) OR TOPIC: (Fibrillation, Persistent Atrial) OR TOPIC: (Fibrillations, Persistent Atrial) OR TOPIC: (Persistent Atrial Fibrillations) OR TOPIC: (Familial Atrial Fibrillation) OR TOPIC: (Atrial Fibrillation, Familial) OR TOPIC: (Atrial Fibrillations, Familial) OR TOPIC: (Familial Atrial Fibrillations) OR TOPIC: (Fibrillation, Familial Atrial) OR TOPIC: (Fibrillations, Familial Atrial) OR TOPIC: (Paroxysmal Atrial Fibrillation) OR TOPIC: (Atrial Fibrillation, Paroxysmal) OR TOPIC: (Atrial Fibrillations, Paroxysmal) OR TOPIC: (Fibrillation, Paroxysmal Atrial) OR TOPIC: (Paroxysmal Atrial Fibrillations)

# 4 TOPIC: (Atrial Flutter) OR TOPIC: (Atrial Flutters) OR TOPIC: (Flutter, Atrial) OR TOPIC: (Flutters, Atrial) OR TOPIC: (Auricular Flutter) OR TOPIC: (Auricular Flutters) OR TOPIC: (Flutter, Auricular) OR TOPIC: (Flutters, Auricular) OR TOPIC: (atrial fibrillation*) OR TOPIC: (atrial flutter*) OR TOPIC: (auricular fibrillation*)

# 5 #2 OR #1

# 6 #4 OR #3

# 7 #6 AND #5

1. **Embase**

#1. 'chronic obstructive lung disease'/exp

#2. 'chronic airflow obstruction':ab,ti OR 'chronic airway obstruction':ab,ti OR 'chronic obstructive bronchitis':ab,ti OR 'chronic obstructive bronchopulmonary disease':ab,ti OR 'chronic obstructive lung disorder':ab,ti OR 'chronic obstructive pulmonary disease':ab,ti OR 'chronic obstructive pulmonary disorder':ab,ti OR 'chronic obstructive respiratory disease':ab,ti OR copd:ab,ti OR 'lung chronic obstructive disease':ab,ti OR 'lung disease, chronic obstructive':ab,ti OR 'lung diseases, obstructive':ab,ti OR 'obstructive lung disease':ab,ti OR 'obstructive lung disease, chronic':ab,ti OR 'obstructive pulmonary disease':ab,ti OR 'obstructive respiratory disease':ab,ti OR 'obstructive respiratory tract disease':ab,ti OR 'pulmonary disease, chronic obstructive':ab,ti OR 'pulmonary disorder, chronic obstructive':ab,ti

#3. 'lung emphysema'/exp

#4. 'bulbous emphysema':ab,ti OR 'bullous emphysema':ab,ti OR 'centrilobular emphysema':ab,ti OR 'chronic lung emphysema':ab,ti OR 'emphysema pulmonale':ab,ti OR 'emphysema, bullous':ab,ti OR 'intrapulmonary interstitial emphysema':ab,ti OR 'lobular emphysema':ab,ti OR 'lung bullous emphysema':ab,ti OR 'lung interstitial emphysema':ab,ti OR 'panacinar emphysema':ab,ti OR 'pneumatosis pulmonum':ab,ti OR pneumonectasia:ab,ti OR 'pulmonary emphysema':ab,ti OR 'unilateral pulmonary emphysema':ab,ti OR 'volumen pulmonum auctum':ab,ti

#5. 'chronic bronchitis'/exp

#6. 'bronchitis chronica':ab,ti OR 'bronchitis, chronic':ab,ti OR 'chronic bronchus infection':ab,ti

#7. #1 OR #2 OR #3 OR #4 OR #5 OR #6

#8. 'new-onset atrial fibrillation'/exp

#9. 'acute atrial fibrillation':ab,ti OR 'acute heart atrium fibrillation':ab,ti OR 'recent-onset atrial fibrillation':ab,ti

#10. 'heart atrium flutter'/exp

#11. 'atrial flutter':ab,ti OR 'atrium flutter':ab,ti OR 'atrium flutter, heart':ab,ti OR 'auricular flutter':ab,ti OR 'cardiac atrial flutter':ab,ti OR 'cardiac atrium flutter':ab,ti OR 'flutter, heart atrium':ab,ti OR 'heart atrial flutter':ab,ti OR 'supraventricular flutter':ab,ti

#12. 'atrial fibrillation'/exp

#13. 'atrium fibrillation':ab,ti OR 'auricular fibrilation':ab,ti OR 'auricular fibrillation':ab,ti OR 'cardiac atrial fibrillation':ab,ti OR 'cardiac atrium fibrillation':ab,ti OR 'fibrillation, heart atrium':ab,ti OR 'heart atrial fibrillation':ab,ti OR 'heart atrium fibrillation':ab,ti OR 'heart fibrillation atrium':ab,ti OR 'non-valvular atrial fibrillation':ab,ti OR 'nonvalvular atrial fibrillation':ab,ti

#14. 'atrial fibrillation*':ab,ti OR 'atrial flutter*':ab,ti OR 'auricular fibrillation*':ab,ti

#15. #8 OR #9 OR #10 OR #11 OR #12 OR #13 OR #14

#16. #7 AND #15

**Appendix 2.** The methodological quality and risk of bias

Table S1. The Newcastle-Ottawa Scale (NOS) for Cohort studies

| Inclusion studies | | Selection | | | | Comparability | Outcome | | Score | |  |
| --- | --- | --- | --- | --- | --- | --- | --- | --- | --- | --- | --- |
| Study | Year | ① | ② | ③ | ④ | ⑤ | ⑥ | ⑦ | ⑧ | - |  |
| Rupak Desai | 2019 | 1 | 1 | 1 | 1 | 1 | 1 | 1 | 1 | 8 |  |
| Xiaochun Xiao | 2019 | 1 | 1 | 1 | 1 | 1 | 1 | 1 | 1 | 8 |  |
| Ya-Hui Wang | 2018 | 1 | 1 | 1 | 1 | 1 | 1 | 1 | 1 | 8 |  |
| Atsushi Hirayama | | 2018 | 1 | 1 | 1 | 1 | 0 | 1 | 1 | 1 | 7 |
| Chung-Yu Chen | 2018 | 1 | 1 | 1 | 1 | 1 | 1 | 1 | 1 | 8 |  |
| J.P. Alves Guimaraes | 2018 | 1 | 1 | 1 | 1 | 1 | 1 | 1 | 1 | 8 |  |
| Kuang Ming Liao | 2017 | 1 | 1 | 1 | 0 | 1 | 1 | 1 | 1 | 7 |  |
| Tomasz Rusinowicz | 2017 | 1 | 0 | 1 | 1 | 0 | 1 | 1 | 1 | 6 |  |
| Wei-Syun Hu | 2017 | 1 | 1 | 1 | 1 | 1 | 1 | 1 | 0 | 7 |  |
| E.A.V. Volchkova | 2015 | 1 | 1 | 0 | 1 | 1 | 1 | 1 | 1 | 7 |  |
| Liza Genao | 2015 | 1 | 1 | 1 | 1 | 1 | 0 | 1 | 1 | 7 |  |
| Jukka Koskela | 2014 | 1 | 1 | 1 | 0 | 1 | 1 | 1 | 1 | 8 |  |
| Harsha V. Ganga | 2013 | 1 | 1 | 1 | 1 | 1 | 1 | 0 | 1 | 7 |  |
| PM Short | 2012 | 1 | 1 | 1 | 1 | 1 | 1 | 1 | 1 | 8 |  |
| Miriam J Warnier | 2010 | 1 | 1 | 1 | 1 | 1 | 1 | 1 | 1 | 8 |  |
| Mitja Lainscak | 2009 | 1 | 1 | 0 | 1 | 1 | 1 | 1 | 1 | 7 |  |

① Representativeness of the exposed cohort

② Selection of the non-exposed cohort

③ Ascertainment of exposure

④ Demonstration that outcome of interest was not present at start of study

⑤ Comparability of cohorts on the basis of the design or analysis

⑥ Assessment of outcome

⑦ Was follow-up long enough for outcomes to occur

⑧ Adequacy of follow up of cohorts

Table S2. The Newcastle-Ottawa Scale (NOS) for Case-control studies

| Inclusion studies | | Selection | | | | Comparability | Outcome | | Score | |
| --- | --- | --- | --- | --- | --- | --- | --- | --- | --- | --- |
| Study | Year | ① | ② | ③ | ④ | ⑤ | ⑥ | ⑦ | ⑧ | - |
| Tomoko Tomioka | 2019 | 1 | 1 | 1 | 1 | 1 | 1 | 1 | 1 | 8 |
| Rashid Nadeem | 2015 | 1 | 1 | 1 | 1 | 0 | 1 | 1 | 1 | 7 |
| Machelle Wilchesky | 2012 | 1 | 1 | 0 | 0 | 1 | 1 | 1 | 1 | 6 |
| Bartolome Celli | 2010 | 1 | 1 | 1 | 1 | 1 | 1 | 1 | 1 | 8 |

①Is the case definition adequate?

②Representativeness of the cases

③Selection of Controls

④Definition of Controls

⑤Comparability of cases and controls on the basis of the design or analysis

⑥Ascertainment of exposure

⑦Same method of ascertainment for cases and controls

⑧Non-Response rate

**Appendix 3.** Sensitivity analysis


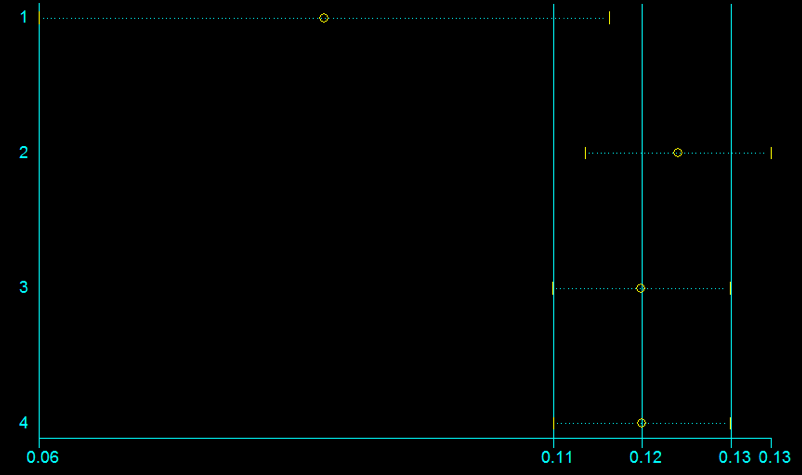


**Figure S1.** The sensitivity analysis for acute respiratory failure

1, the study by Rupak Desai et al.; 2, the study by Xiaochun Xiao et al.; 3, the study by CHUNG-YU CHEN et al.; 4, the study by Tomasz Rusinowicz et al.

**Note:** From the Figure S1, the sensitivity analysis shows that the data from the study by Desai et al. (1) was significant different from other three studies (including Xiao et al., CHEN et al. and Rusinowicz et al.). The data from other three studies was pooled by meta-analysis, and the OR was 1.09 (95% CI: 1.06–1.12) and *I^2^* was 0%. Thus, when the study by Desai et al. was excluded from the analysis, *I^2^* decreased from 57% to 0% and the OR decreased from 1.13 (95% CI: 1.12–1.15) to 1.09 (95% CI: 1.06–1.12).


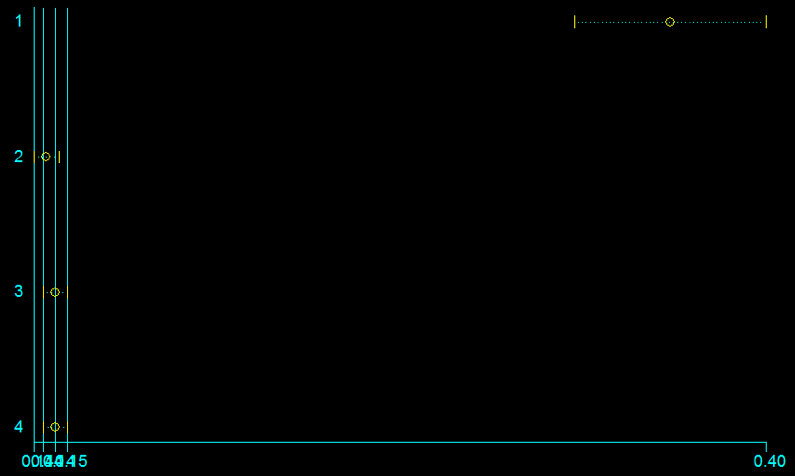


**Figure S2.** The sensitivity analysis for myocardial infarction

1, the study by Rupak Desai et al.; 2, the study by Xiaochun Xiao et al.; 3, the study by J.P. Alves Guimaraes et al.; 4, the study by PM Short et al.

**Note:** From the Figure S2, the sensitivity analysis shows that the data from the study by Desai et al. (1) was significant different from other three studies (including Xiao et al., Guimaraes et al. and Short et al.). The data from other three studies was pooled by meta-analysis, and the OR was 1.55 (95% CI: 1.29–1.85) and *I^2^* was 63%. Thus, when the study by Desai et al. was excluded from the analysis, *I^2^* decreased from 98% to 63% and the OR increased from 1.41 (95% CI: 1.19–1.69) to 1.55 (95% CI: 1.29–1.85).


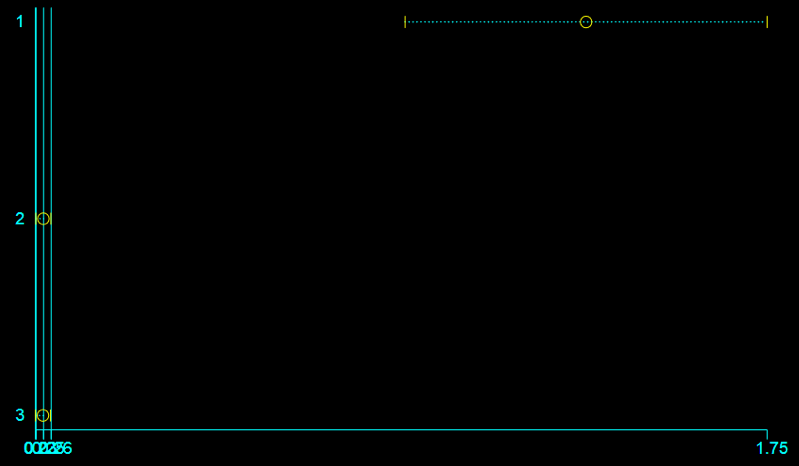


**Figure S3.** The sensitivity analysis for coronary artery disease

1, the study by Rupak Desai et al.; 2, the study by E.A.V. Volchkova et al.; 3, the study by PM Short et al.

**Note:** From the Figure S3, the sensitivity analysis shows that the data from the study by Desai et al. (1) was significant different from other two studies (including Volchkova et al. and Short et al.). The data from other two studies was pooled by meta-analysis, and the OR was 3.88 (95% CI: 2.43–6.21) and *I^2^* was 35%. Thus, when the study by Desai et al. was excluded from the analysis, *I^2^* decreased from 94% to 35% and the OR increased from 2.57 (95% CI: 1.01–6.49) to 3.88 (95% CI: 2.43–6.21).
