# Supplementary material for: Risk factors for new-onset atrial fibrillation in patients with chronic obstructive pulmonary disease: a systematic review and meta-analysis
Source: PeerJ. 2020 Dec 2;8:e10376. doi: 10.7717/peerj.10376 (PMC7718784; doi:10.7717/peerj.10376)
Supplement: Supplemental Information 2 [file peerj-08-10376-s002.doc]

| **Section/topic** | **#** | **Checklist item** | **Reported on page #** |
| --- | --- | --- | --- |
| **TITLE** | | |  |
| Title | 1 | Identify the report as a systematic review, meta-analysis, or both. | Page 1: Risk factors for new-onset atrial fibrillation in patients with chronic obstructive pulmonary disease: a systematic review and meta-analysis |
| **ABSTRACT** | | |  |
| Structured summary | 2 | Provide a structured summary including, as applicable: background; objectives; data sources; study eligibility criteria, participants, and interventions; study appraisal and synthesis methods; results; limitations; conclusions and implications of key findings; systematic review registration number. | Page 3: **Background.** New-onset atrial fibrillation (AF) in patients with chronic obstructive pulmonary disease (COPD) is associated with an accelerated decline in lung function, and a significant increase in mortality rate. A deeper understanding of the risk factors for new-onset AF during COPD will provide insights into the relationship between COPD and AF and guide clinical practice. This systematic review and meta-analysis is designed to identify risk factors for new-onset AF in patients with COPD, and to formulate recommendations for preventing AF in COPD patients that will assist clinical decision making.  **Methods.** PubMed, Embase, Web of Science and Cochrane Library databases were searched for studies, which reported the results of potential risk factors for new-onset AF in COPD patients.  **Results.** Twenty studies involving 8,072,043 participants were included. Fifty factors were examined as potential risk factors for new-onset AF during COPD. Risk factors were grouped according to demographics, comorbid conditions, and COPD- and cardiovascular-related factors. In quantitative analysis, cardiovascular- and demographic-related factors with a greater than 50% change in the odds of new-onset AF, including age, acute care encounter, coronary artery disease, heart failure and congestive heart failure. In qualitative analysis, the comparison of the risk factors was conducted between COPD-associated AF and non-COPD-associated AF. Cardiovascular-related factors for non-COPD-associated AF were also considered as risk factors for new-onset AF during COPD; however, the influence tended to be stronger during COPD. In addition, comorbid factors identified in non-COPD-associated AF were not associated with an increased risk of AF during COPD.  **Conclusions.** New-onset AF in COPD has significant demographic characteristics. Older age (over 65 years), males and white race are at higher risk of developing AF. COPD patients with a history of cardiovascular disease should be carefully monitored for new-onset of AF, and appropriate preventive measures should be implemented. Even patients with mild COPD are at high risk of new-onset AF. This study shows that risk factors for new-onset AF during COPD are mainly those associated with the cardiovascular-related event and are not synonymous with comorbid factors for non-COPD-associated AF. The pathogenesis of COPD-associated AF may be predominantly related to the cardiac dysfunction caused by the chronic duration of COPD, which increases the risk of cardiovascular-related factors and further increases the risk of AF during COPD.  **PROSPERO registration number.** CRD42019137758 |
| **INTRODUCTION** | | |  |
| Rationale | 3 | Describe the rationale for the review in the context of what is already known. | Page 4-5: Chronic obstructive pulmonary disease (COPD) is the fourth leading cause of morbidity and mortality worldwide^1^, and is predicted to rank third by 2030 ^2^. Atrial fibrillation (AF) often has a negative impact on patients with chronic disease, and is the most common arrhythmia in patients with COPD^3^. The co-existence of COPD and AF is associated with mutual exacerbation of the conditions, which complicates the management in clinical practice^4^. New-onset AF in patients with COPD is associated with an accelerated decline in lung function, and a significant increase in cardiovascular accidents and long-term mortality^5^. For patients with COPD, the worldwide prevalence of new-onset AF is as high as 14.3%, and for patients over 75 years old, the prevalence rises to 40% ^6^.  COPD is one of the main risk factors for AF. For patients with COPD, the high incidence of new-onset AF is combined with an alarming mortality rate. The incidence of AF in COPD patients is more than four times higher than that in non-COPD patients^7^. The hospital mortality rate for patients with COPD-associated AF is 5.7%, whereas the corresponding rate for non-COPD-associated AF patients is 2.2% ^6, 7^. Although the prevalence and short-term mortality rate of new-onset AF in COPD patients are high, the management of cardiovascular conditions in COPD patients is often ignored in current clinical practice. Elucidating the risk factors will provide an effective approach to screening high-risk COPD patients with new-onset AF and improving the clinical course and prognosis of COPD. A deeper understanding of the risk factors for new-onset AF during COPD will also provide insights into the relationship between COPD and AF and guide clinical practice. Based on the mutual promotion of deterioration between COPD and AF and the difference in primary disease settings, the risk factors for AF in the non-COPD setting do not adequately reflect the risk of AF during COPD. However, there is a paucity of information on the incidence of AF and insufficient evidence of risk factors in COPD patients from most studies. |
| Objectives | 4 | Provide an explicit statement of questions being addressed with reference to participants, interventions, comparisons, outcomes, and study design (PICOS). | Page 5: Comprehensive integration of risk factors for AF in patients with COPD is important in bridging the knowledge gaps and providing information for the application of COPD guidelines. Therefore, this systematic review and meta-analysis was designed to identify risk factors for new-onset AF in patients with COPD, and to formulate recommendations for limiting AF in COPD patients that will assist clinical decision making. |
| **METHODS** | | |  |
| Protocol and registration | 5 | Indicate if a review protocol exists, if and where it can be accessed (e.g., Web address), and, if available, provide registration information including registration number. | Page 5: All methods of this systematic reviews and meta-analyses followed the referred Reporting Items for Systematic Reviews and Meta-Analyses (PRISMA) guidelines ^8^. Details of the preregistered protocol for this study are available on International prospective register of systematic reviews (PROSPERO; CRD42019137758) |
| Eligibility criteria | 6 | Specify study characteristics (e.g., PICOS, length of follow-up) and report characteristics (e.g., years considered, language, publication status) used as criteria for eligibility, giving rationale. | Page 6: This meta-analysis included studies that met the following inclusion criteria:  1. Adult patients diagnosed with COPD (18 years of age or older). COPD was diagnosed according to the latest reference standards during the study, such as the GOLD criteria.  2. The studies reported the results of identified risk factors for new-onset AF in COPD patients. The adjusted odds ratio (OR), SE, and 95% confidence intervals (CI) for each candidate risk factor are reported as appropriate.  3. No publication date, status or language restrictions were applied.  Exclusion criteria:  1. Secondary study, editorials, and animal experiments were excluded.  2. Studies that included only unadjusted OR, studies that did not separate paroxysmal AF from new-onset AF, and studies that involved new-onset AF in patients only after cardiothoracic surgery. |
| Information sources | 7 | Describe all information sources (e.g., databases with dates of coverage, contact with study authors to identify additional studies) in the search and date last searched. | Page 5: The review authors searched for medical literature before December 2019. The search was conducted in four electronic databases including the Cochrane Library, PubMed, Embase, Web of Science (WOS), and the reference lists from review articles. |
| Search | 8 | Present full electronic search strategy for at least one database, including any limits used, such that it could be repeated. | Page 6: The search strategy used a combination of MeSH, Emtree and text word, conducted with the following keywords: COPD or chronic obstructive pulmonary disease and AF or atrial fibrillation. The review articles with relevant topic were screened through keyword search, and their reference lists were further searched for eligible studies. The details of search strategies can be found in the Supplementary (Appendix 1. Search Strategy). |
| Study selection | 9 | State the process for selecting studies (i.e., screening, eligibility, included in systematic review, and, if applicable, included in the meta-analysis). | Page 6-7: Two authors (T Shuai, C Zhang) independently reviewed the studies to be included in light of the titles, abstracts and keywords. If a study was found relevant to this topic, the full text would be further evaluated by at least two reviewers to assess whether it fulfills the selection criteria. In case of inconsistency between the reviewers, the third reviewer (J Liu) would be consulted. An intra-rater reliability was conducted to assess the consistency of the study selection between two independent review authors. A study diagram was prepared for this selection to demonstrate the entire process of literature research and the selection of studies. |
| Data collection process | 10 | Describe method of data extraction from reports (e.g., piloted forms, independently, in duplicate) and any processes for obtaining and confirming data from investigators. | Page 7: The data was independently extracted by two review authors (Q Huang and H Xiong), and the resulting differences were resolved by a third reviewer (J Liu). The extracted data included: 1) citation information, participant characteristics, study types, and study settings; 2) definitions of COPD and AF; and 3) the adjusted OR, SE, and 95% CI for each candidate risk factor. If the data were insufficient, an email was sent to the authors to request the data. |
| Data items | 11 | List and define all variables for which data were sought (e.g., PICOS, funding sources) and any assumptions and simplifications made. | Page 7: The extracted data included: 1) citation information, participant characteristics, study types, and study settings; 2) definitions of COPD and AF; and 3) the adjusted OR, SE, and 95% CI for each candidate risk factor. If the data were insufficient, an email was sent to the authors to request the data. |
| Risk of bias in individual studies | 12 | Describe methods used for assessing risk of bias of individual studies (including specification of whether this was done at the study or outcome level), and how this information is to be used in any data synthesis. | Page 7 and Tables S1 and S2: Two review authors (J Liu and J Lu) independently applied the guidelines of the PRISMA statement to evaluate each involved study ^9^. For observational studies such as case-control and cohort studies, the Newcastle-Ottawa Scale (NOS) was conducted ^10^ to assess the methodological quality and risk of bias (Table S1 and Table S2). In case of any inconsistency, an agreement was reached through discussion between all authors. |
| Summary measures | 13 | State the principal summary measures (e.g., risk ratio, difference in means). | Page 7-8: Extracted data were analyzed using Stata SE 14.0 (Stata Corp; College Station, TX, USA). For the random-effects method, a variation of the inverse-variance method is to incorporate an assumption that different studies are estimating different but related factor effects. Considering the play of chance and some genuine variation in the in risk-factor effects, the pooled effect size was calculated by the random-effects method in this study.  The primary result was a pooled-adjusted OR for each risk factor using a general inverse variance method with random effects model. |
| Synthesis of results | 14 | Describe the methods of handling data and combining results of studies, if done, including measures of consistency (e.g., I^2^) for each meta-analysis. | Page 8: Extracted data were analyzed using Stata SE 14.0 (Stata Corp; College Station, TX, USA). For the random-effects method, a variation of the inverse-variance method is to incorporate an assumption that different studies are estimating different but related factor effects. Considering the play of chance and some genuine variation in the in risk-factor effects, the pooled effect size was calculated by the random-effects method in this study. The heterogeneity of eligible studies was assessed by the Cochrane *Q* test (substantive heterogeneity was indicated by *P* < 0.05) and the *I^2^* test (substantive heterogeneity was indicated by *I^2^* > 50%). If substantive heterogeneity existed, sensitivity analysis was performed to analyze the potential sources of the heterogeneity. Egger’s test was prepared for assessing the publication bias in case that there are over 10 included studies ^11^. The α value was set to 0.05. |

Page 1 of 2

| **Section/topic** | **#** | **Checklist item** | **Reported on page #** |
| --- | --- | --- | --- |
| Risk of bias across studies | 15 | Specify any assessment of risk of bias that may affect the cumulative evidence (e.g., publication bias, selective reporting within studies). | Page 8: The heterogeneity of eligible studies was assessed by the Cochrane *Q* test (substantive heterogeneity was indicated by *P* < 0.05) and the *I^2^* test (substantive heterogeneity was indicated by *I^2^* > 50%). If substantive heterogeneity existed, sensitivity analysis was performed to analyze the potential sources of the heterogeneity. Egger’s test was prepared for assessing the publication bias in case that there are over 10 included studies ^11^. The α value was set to 0.05. |
| Additional analyses | 16 | Describe methods of additional analyses (e.g., sensitivity or subgroup analyses, meta-regression), if done, indicating which were pre-specified. | Page 8: If substantive heterogeneity existed, sensitivity analysis was performed to analyze the potential sources of the heterogeneity. Egger’s test was prepared for assessing the publication bias in case that there are over 10 included studies ^11^. The α value was set to 0.05. |
| **RESULTS** | | |  |
| Study selection | 17 | Give numbers of studies screened, assessed for eligibility, and included in the review, with reasons for exclusions at each stage, ideally with a flow diagram. | Page 9: A flow chart of the study selection process prepared according to the PRISMA guidelines is presented in Figure 1^10^. After reviewing the title and abstract, 150 articles were screened for full-text review. Of these, 130 articles failed to meet the inclusion criteria. Twenty studies were found to fulfill all the criteria and involved 8,072,043 participants. Inter-rater reliability was strong (ĸ, 0.80). |
| Study characteristics | 18 | For each study, present characteristics for which data were extracted (e.g., study size, PICOS, follow-up period) and provide the citations. | Page 9: Of the 20 included studies^15-34^, 13 studies used retrospective cohort designs^15, 16, 18, 20-24, 27, 28, 32-34^, three were prospective cohorts ^19, 26, 30^, three were case-control studies ^17, 25, 31^, and one was a prospective nested case-control study ^29^. All studies examined new-onset AF in patients with COPD. A summary of the characteristics of the included studies is shown in Table 1. |
| Risk of bias within studies | 19 | Present data on risk of bias of each study and, if available, any outcome level assessment (see item 12). | Page 10: As shown in Table 1, the methodological quality of the observational studies was rated high, with 10 studies scoring nine of nine on the NOS ^15-18, 20, 21, 30-32, 34^, six scoring eight of nine ^19, 24-27, 33^, three scoring seven of nine ^22, 28, 29^, and scoring six of nine ^23^. The overall risk of bias was rated low; however, the included studies used different disease definitions, which influenced the population selection. Summaries of the risk of bias are shown in Tables S1 and S2 (Supplementary file; Appendix 2). |
| Results of individual studies | 20 | For all outcomes considered (benefits or harms), present, for each study: (a) simple summary data for each intervention group (b) effect estimates and confidence intervals, ideally with a forest plot. | Page 10: Figure 2 shows the effect sizes of all 50 factors (un-pooled and pooled) examined as potential risk factors for new-onset AF during COPD. Twenty-five factors presented in at least two studies were included in the pooled meta-analysis. Eighteen pooled factors were associated with increased odds of new-onset AF: age (over 65 years and over 75 years), male, urban population, any acute care encounter, hyperlipidemia, sepsis, renal failure, pneumonia, acute respiratory failure, invasive mechanical ventilation (IMV), noninvasive mechanical ventilation (NMV), ipratropium bromide use, short-acting β-agonist use, myocardial infarction (MI), coronary artery disease (CAD), heart failure (HF) and congestive heart failure (CHF). Of these, six factors were associated with a greater than 50% increase in odds: age (over 65 years and over 75 years), acute care encounter, CAD, HF and CHF. |
| Synthesis of results | 21 | Present results of each meta-analysis done, including confidence intervals and measures of consistency. | Page 10-11: Figure 2 shows the effect sizes of all 50 factors (un-pooled and pooled) examined as potential risk factors for new-onset AF during COPD. Twenty-five factors presented in at least two studies were included in the pooled meta-analysis. Eighteen pooled factors were associated with increased odds of new-onset AF: age (over 65 years and over 75 years), male, urban population, any acute care encounter, hyperlipidemia, sepsis, renal failure, pneumonia, acute respiratory failure, invasive mechanical ventilation (IMV), noninvasive mechanical ventilation (NMV), ipratropium bromide use, short-acting β-agonist use, myocardial infarction (MI), coronary artery disease (CAD), heart failure (HF) and congestive heart failure (CHF). Of these, six factors were associated with a greater than 50% increase in odds: age (over 65 years and over 75 years), acute care encounter, CAD, HF and CHF.  Two pooled factors were associated with decreased odds of new-onset AF in the meta-analysis: black ethnicity (compared with white), and peripheral vascular disease (PVD). Five factors were not associated with new-onset AF after the pooled analysis: hypertension, diabetes mellitus (DM), hepatic failure, long-acting β-agonist use and theophylline use. |
| Risk of bias across studies | 22 | Present results of any assessment of risk of bias across studies (see Item 15). | Page 10 and Tables S1 and S2: As shown in Table 1, the methodological quality of the observational studies was rated high, with 10 studies scoring nine of nine on the NOS ^15-18, 20, 21, 30-32, 34^, six scoring eight of nine ^19, 24-27, 33^, three scoring seven of nine ^22, 28, 29^, and scoring six of nine ^23^. The overall risk of bias was rated low; however, the included studies used different disease definitions, which influenced the population selection. Summaries of the risk of bias are shown in Tables S1 and S2 (Supplementary file; Appendix 2). |
| Additional analysis | 23 | Give results of additional analyses, if done (e.g., sensitivity or subgroup analyses, meta-regression [see Item 16]). | Page 11: Sensitivity analysis of acute respiratory failure, MI and CAD showed that the high heterogeneity was derived from the study by Desai et al.^15^. The results of sensitivity analysis are shown in Supplementary Figures S1, S2, and S3 (Appendix 3. Sensitivity analysis). When the study by Desai et al. ^15^ was excluded from the analysis, *I^2^* decreased to 0% and the OR decreased from 1.12 to 1.09 (95% CI: 1.06–1.12) for acute respiratory failure, *I^2^* decreased from 98% to 63% and the OR increased to 1.55 (95% CI: 1.29–1.85) for MI and *I^2^* decreased from 94% to 35% and the OR increased to 3.88 (95% CI: 2.43–6.21) for CAD. |
| **DISCUSSION** | | |  |
| Summary of evidence | 24 | Summarize the main findings including the strength of evidence for each main outcome; consider their relevance to key groups (e.g., healthcare providers, users, and policy makers). | Page 13: In this, study, we conducted a systematic review and meta-analysis to characterize and evaluate risk factors for new-onset AF in COPD patients. Fifty potential risk factors were grouped by factor type. Quantitative analysis demonstrated that risk factors for new-onset AF during COPD are mainly those associated with cardiovascular-related events and demographic factors (e.g. age, acute care encounter, CAD, HF and CHF). Although COPD-related factors can also trigger new-onset AF in COPD patients, the risk effect is moderate. In this study, we further explored the differences between the risk factors for COPD-associated AF and the identified risk factors for non-COPD-associated AF. Although cardiovascular-related factors for non-COPD-associated AF are also considered to be risk factors for new-onset AF during COPD, the influences are stronger in COPD patients. Interestingly, comorbid factors (DM, prior beta-blocker use, hypertension, PVD and hepatic failure) identified in non-COPD-associated AF are not associated with an increased risk of new-onset AF during COPD. This highlights the importance of distinguishing risk factors for AF in patients with COPD and the potential need for different preventive interventions. |
| Limitations | 25 | Discuss limitations at study and outcome level (e.g., risk of bias), and at review-level (e.g., incomplete retrieval of identified research, reporting bias). | Page 17-18: There are several limitations in the current systematic review and meta-analysis. First, due to the substantive heterogeneity, we performed a sensitivity analysis to explore the source of heterogeneity. We identified the study by Desai et al. 15 as the major source of heterogeneity. The analysis of heterogeneity is limited due to the insufficient number of studies reporting risk factors. In addition, other potential sources of heterogeneity in our analysis of the results of baseline characteristics are as follows: 1) The designs of the included studies were heterogeneous. Compared with meta-analyses including randomized controlled trials, those including observational studies have greater potential bias. 2) The studies included patients with diseases defined using different strategies. Although all strategies are reasonable, different criteria may lead to variations in clinicians’ decision-making, which may increase the heterogeneity of population selection.  Second, interpretation of the observed ORs depends on all necessary confounders being included, without any colliders or intermediate variables. However, the adjusted ORs reported in the included studies cannot eliminate residual confounding caused by unknown, missed and inaccurate confounding factors, and there is a lack of discussion about the causality of the variables. Therefore, limited by the existing methods, it is difficult to analyze the causal associations of 50 variables without additional original research. Furthermore, we also required more original research to use the directed acyclic graph to distinguish the confider, collider, and mediator in risk factors. Third, because no risk factor was reported in 10 or more original studies; Egger’s test and funnel plots were not included in our analysis. Further high-quality original studies are still required to limit the potential publication bias. |
| Conclusions | 26 | Provide a general interpretation of the results in the context of other evidence, and implications for future research. | Page 18: This systematic review and meta-analysis reveals the demographic characteristics of patients with new-onset AF during COPD. Older age (over 65 years), males and whites are at higher risk of developing AF. The dominant factors are related to cardiovascular (CAD, HF and CHF), and may be amplified in the context of COPD. COPD patients with a history of cardiovascular disease should be carefully monitored for new-onset AF, and appropriate preventive measure should be implemented. Even for patients with mild COPD, clinicians should not relax their vigilance in monitoring patients for new-onset AF. The pathogenesis of AF during COPD may be primarily related to cardiac dysfunction caused by the chronic duration of COPD, which increases the risk effect of cardiovascular-related factors and further increases the risk of AF in patients with COPD. This highlights the importance of identifying risk factors for predicting COPD-associated AF and the potential need for different preventive interventions. |
| **FUNDING** | | |  |
| Funding | 27 | Describe sources of funding for the systematic review and other support (e.g., supply of data); role of funders for the systematic review. | Page 18: The authors remain independently of any funding influence. |

*From:*  Moher D, Liberati A, Tetzlaff J, Altman DG, The PRISMA Group (2009). Preferred Reporting Items for Systematic Reviews and Meta-Analyses: The PRISMA Statement. PLoS Med 6(7): e1000097. doi:10.1371/journal.pmed1000097

For more information, visit: **www.prisma-statement.org**.

Page 2 of 2
