# Supplementary material for: Risk factors for new-onset atrial fibrillation in patients with chronic obstructive pulmonary disease: a systematic review and meta-analysis
Source: PeerJ. 2020 Dec 2;8:e10376. doi: 10.7717/peerj.10376 (PMC7718784; doi:10.7717/peerj.10376)
Supplement: Supplemental Information 3 [file peerj-08-10376-s003.docx]

**The rationale for conducting the meta-analysis**

Atrial fibrillation (AF) often has a negative impact on patients with chronic disease, and is the most common arrhythmia in patients with chronic obstructive pulmonary disease (COPD). The co-existence of COPD and AF exacerbates each other, complicating the clinical practice course. New-onset AF in patients with COPD is associated with an accelerated decline in lung function, and a significant increase in cardiovascular accidents and mortality rate.

The incidence rates of AF in COPD patients was more than 4 times higher than in non-COPD patients. As for in-hospital mortality rate, patients with COPD associated AF is 5.7%, whereas non-COPD associated AF patients is 2.2%. Although the prevalence and the short-term mortality of new-onset AF in COPD patients are high, the current management of cardiovascular conditions is often ignored in COPD clinical practice.

Elucidating the risk factors will provide a guarantee for screening high-risk COPD patients and improving the clinical course and prognosis of COPD. A deeper understanding of the risk factors for new-onset AF during COPD can also provide insight into the relationship between COPD and AF and guide clinical practice. Based on the mutual promotion of deterioration between COPD and AF and the difference in primary disease settings, the risk factors of AF in the non-COPD setting are not suitable to reflect the AF risks during COPD. However, there is a paucity of information on AF incidence and insufficient evidence of risk factors in COPD patients in most studies.

Therefore, this systematic review and meta-analysis is designed to identify risk factors for new-onset AF in patients with COPD, and to propose recommendations for limiting AF in COPD patients and to assist clinical decision making.

**The contribution that the meta-analysis makes to knowledge in light of previously published related reports, including other meta-analyses and systematic reviews.**

In previously published related reports, most of emerging evidences only evaluated specific single risk factors such as hyperlipidemia, sepsis, pneumonia or coronary heart disease (CAD), which is difficult to comprehensive demonstrate the risk factors of new-onset AF in patients with COPD. Based on these available evidences, it is not only inefficient to assess and compare the risk level of potential factors, but also limits the generalizability of evaluating risk factors for COPD-associated AF.

In this study, we conducted a comprehensive evidence-based study on the risk factors of new-onset AF in patients with COPD, and evaluated a total of 50 factors as potential risk factors, including 25 factors of quantitative analysis and 25 factors of qualitative analysis. Potential risk factors were grouped by factor type. Quantitative analysis demonstrated that the strongest factors were related to cardiovascular and demographic factors (e.g. age, acute care encounter, CAD, HF and CHF). Although COPD-related factors can also trigger new-onset AF during COPD, the risk effect is moderate. Interestingly, co-morbid factors (DM, prior beta blocker use, hypertension, PVD and hepatic failure) are not associated with an increased risk of new-onset AF during COPD.

Previous studies did not distinguish the difference of risk factors between COPD-associated AF and non-COPD-associated AF. However, based on the mutual promotion of deterioration between COPD and AF, and the difference in primary disease settings, the risk factors of AF in the non-COPD setting are not suitable to reflect the AF risks during COPD. In most studies, there is a lack of information on AF incidence and insufficient evidence of risk factors in COPD patients.

Therefore, in this study we further explored the differences between the risk factors of COPD-associated AF and the identified risk factors of non-COPD-associated AF. We found that although cardiovascular related factors of non-COPD-associated AF are also considered as risk factors for new-onset AF during COPD, the effect in COPD patients is stronger. In addition, co-morbid factors (DM, prior beta blocker use, hypertension, PVD and hepatic failure) identified in non-COPD-associated AF are not associated with increased risk of new-onset AF during COPD. Given these concerns, the importance of distinguishing risk factors for AF in patients with COPD and the potential need for different preventive interventions are warranted.

This systematic review and meta-analysis elucidates that new-onset AF during COPD has significant demographic characteristics. Older age (over 65 years of age), men and whites are at higher risk of developing AF. The dominant factors are related to cardiovascular (CAD, HF and CHF), and may be amplified under the COPD setting. For COPD patients with history of cardiovascular diseases, importance should be attached to the new-onset of AF, and appropriate preventive measures should be taken. Even for patients with mild COPD, clinicians should not relax their vigilance against new-onset AF. In addition, the pathogenesis of AF during COPD may primarily relate to cardiac dysfunction caused by the chronic duration of COPD, which increases the risk effect of cardiovascular-related factors and further increases the risk of AF during COPD.
